# Lipids at the Nexus between Cerebrovascular Disease and Vascular Dementia: The Impact of HDL-Cholesterol and Ceramides

**DOI:** 10.3390/ijms24054403

**Published:** 2023-02-23

**Authors:** Domenico Sergi, Enrico Zauli, Veronica Tisato, Paola Secchiero, Giorgio Zauli, Carlo Cervellati

**Affiliations:** 1 Department of Translational Medicine, University of Ferrara, 44121 Ferrara, Italy; 2 Department of Translational Medicine and LTTA Centre, University of Ferrara, 44121 Ferrara, Italy; 3 King Khaled Eye Specialistic Hospital, Riyadh 11462, Saudi Arabia

**Keywords:** cerebrovascular disease, vascular dementia, HDL-cholesterol, ceramides, saturated fatty acids, omega-3 fatty acids

## Abstract

Cerebrovascular diseases and the subsequent brain hypoperfusion are at the basis of vascular dementia. Dyslipidemia, marked by an increase in circulating levels of triglycerides and LDL-cholesterol and a parallel decrease in HDL-cholesterol, in turn, is pivotal in promoting atherosclerosis which represents a common feature of cardiovascular and cerebrovascular diseases. In this regard, HDL-cholesterol has traditionally been considered as being protective from a cardiovascular and a cerebrovascular prospective. However, emerging evidence suggests that their quality and functionality play a more prominent role than their circulating levels in shaping cardiovascular health and possibly cognitive function. Furthermore, the quality of lipids embedded in circulating lipoproteins represents another key discriminant in modulating cardiovascular disease, with ceramides being proposed as a novel risk factor for atherosclerosis. This review highlights the role of HDL lipoprotein and ceramides in cerebrovascular diseases and the repercussion on vascular dementia. Additionally, the manuscript provides an up-to-date picture of the impact of saturated and omega-3 fatty acids on HDL circulating levels, functionality and ceramide metabolism.

## 1. Introduction

The developed and, increasingly, the developing countries are facing an epidemic of obesity which is posing an enormous burden to people’s health along with a huge economical pressure to the healthcare systems worldwide. Apart from representing a risk factor for a plethora of diseases, including type 2 diabetes and certain types of cancer [1], obesity and its cardio-metabolic aberrations have also been associated with neurodegenerative diseases [2], including several types of dementia [3]. Remarkably, obesity also increases the risk of developing cardiovascular disease (CAD), also encompassing cerebrovascular disease (CVD), which, in turn, is pivotal in the pathogenesis of vascular dementia (VaD). 

Dyslipidemia, referred to as an increase in LDL-cholesterol as well as triglycerides and a decrease in HDL-cholesterol, represents a key pathogenetic driver of CAD and CVD. In this context, HDL lipoproteins have generally been considered as beneficial for cardiovascular health [4]. However, increasing their circulating levels was not sufficient to prevent CAD [5], which supports the notion that their circulating levels are not the only discriminant in preventing cardiovascular and cerebrovascular events. Indeed, the quality and functionality of HDL lipoproteins, including their cholesterol efflux capacity, anti-oxidant and anti-inflammatory properties, appear to be more relevant than HDL-cholesterol plasma levels in dictating the protective effects of these lipoproteins on cardiovascular and cerebrovascular health [6]. Additionally, advances in omics techniques and particularly in lipidomics have allowed the identification of other risk factors for CAD besides the classical circulating lipid profile encompassing triglycerides, LDL and HDL cholesterol. In this regard, ceramides emerged as a pivotal driver of CAD in light of their ability to contribute to atherosclerosis and trigger inflammatory responses [7,8,9,10].

Importantly, the quality of dietary fatty acids is a key discriminant in shaping CAD and consequently CVD [11,12], an effect which also relates to the ability of different dietary lipids to shape HLD-cholesterol circulating levels [13], quality, functionality [14] and to modulate ceramide synthesis [15,16].

Thus, the aim of this review is to provide an overview of the role of HDL lipoproteins and ceramides, and their modulation by dietary fatty acids, on CVDs and the potential repercussions on VaD.

## 2. VaD

**Figure 1:**
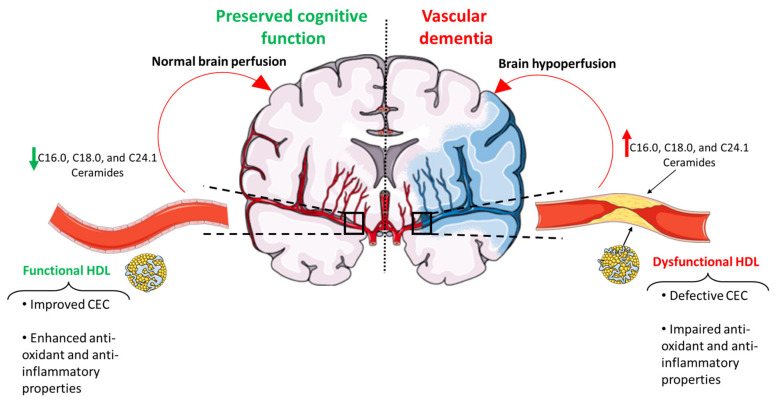
Impact of HDL lipoprotein functionality and ceramides on VaD. An impairment in HDL lipoprotein cholesterol efflux capacity, anti-oxidant and anti-inflammatory properties, in concert with the upregulation of C16.0, C18.0 and C24.1 ceramides promotes atherosclerosis, which is pivotal in promoting CVDs and impairing brain perfusion. The latter, in turn, represents a key pathogenetic factor leading to VaD. CEC: cholesterol efflux capacity. This figure was created using smart.servier.com.

VaD is a neurocognitive disorder, which represents the most frequent form of dementia after Alzheimer’s disease (AD), accounting for 20% of all cases [17]. Currently, this is a major health issue, since there are almost 57 million people affected by dementia, with a prevalence that will increase to 152 million in 2050 [18]. The most common symptoms of VaD are memory decline, early gait disorder, depression and personality changes. The cognitive impairment characterizing this disease results from brain hypoperfusion due to cerebrovascular diseases (CVDs) (Figure 1). In turn, CVD is a wide and heterogeneous category of disorders including a wide array of vascular damage and dysfunction. Stroke is by far the foremost type of CVD, and its occurrence increases the risk of VaD two-fold [19]. Other initially covert neurovascular lesions can contribute to VaD, such as white matter hyperintensities, cerebral microbleeds and microinfarcts. 

These vascular abnormalities are often caused by cerebral amyloid angiopathy, a common age-related small vessel disease. This type of microangiopathy is the result of amyloid-β deposition, preferentially involving cortical and leptomeningeal small and medium vessels. Cerebral amyloid angiopathy causes changes in brain parenchyma, in the blood–brain barrier (BBB) integrity, promotes the release of inflammatory mediators, induces matrix metalloproteases and free radicals and exacerbates brain hypoperfusion. Considering its ability to induce the aforementioned pathogenetic factors, it is not surprising that cerebral amyloid angiopathy is strongly associated with the risk of dementia in elderly people. Indeed, it is a very common neuropathological feature in VaD, but also AD.

## 3. Dyslipidemia: A Non-Exhaustive Definition

Dyslipidemia is conventionally referred to as an unbalance in the levels of cholesterol, low-density lipoprotein cholesterol (LDL-C), triglycerides and high-density lipoprotein cholesterol (HDL-C). This condition, along with hypertension, is now referred to as the foremost risk factor for CAD and CVDs.

Despite the enormous amount of epidemiological and clinical data in support of the use of plasma lipoprotein and lipid levels as predictive biomarkers and pharmacological targets in all vascular pathologies, there is an ample and increasing awareness that conventional lipid profiles are unable to fully explain CVD and CAD risk. An emblematic example in this frame is given by LDL-C and HDL-C. High plasma levels of LDL-C have been a well-established risk factor for CADs and CVDs, and are still being used as the main biomarker of these pathologies. However, the risk of death in individuals with low LDL-C level is well-documented and not-negligible residual risk remains after achieving the recommended LDL-C targets [20].

Similar considerations apply to HDL-C. Indeed, on one hand, there is a number of consistent reports showing an inverse association between HDL-C levels and CAD, but also neurodegenerative diseases [21,22]. However, on the other hand, genetic and interventional studies raised some doubts on the cause–effect relationship between this lipoprotein, or better, its cholesterol content, and the onset of these pathologies. Several clinical trials have been targeting cholesteryl ester transfer protein, which promotes the transfer of cholesterol from HDL to low density lipoproteins (LDL), very low density lipoprotein (VLDL) and intermediate density lipoprotein (IDL), and triglycerides from these ApoB lipoproteins to HDL, or other HDL-raising agents such as fibrates and niacin. None of them resulted in significant improvements in CAD and CVD prevention [5]. The absence of a causal link between HDL-C and CAD was also confirmed by Mendelian randomization studies; indeed, individuals with genetic polymorphisms associated with high levels of HDL-C did not show any improvement in cardiovascular health [23,24].

The above cited “residual risk”, which is still present in individuals with a well-balanced lipid profile, can have various explanations. The most obvious is the coexistence of subclinical or undiagnosed dysregulated cardiometabolic conditions. Another possibility is that other circulating metabolically active lipids could influence the CVD and CAD risk. As shown in the Mendelian study by Shucheng et al., cholesterol transported in triglyceride-rich lipoproteins (remnant cholesterol) and other remnant lipoproteins can also predict cardiovascular events [20]. Finally, solid data suggest that function, and thus quality, rather than quantity of either particles or lipid content of lipoproteins, might be the best measure to predict CVD and CAD risk. This last aspect is particularly relevant to HDL, which possesses pleiotropic biological function.

## 4. Functional and Dysfunctional HDL

### 4.1. HDL Remodeling

HDL is a heterogeneous class of particles ranging between 7 and 12 nm in diameter and 1.063 and 1.21 g/mL in density [25]. The HDL subtypes differ in terms of shape, density, surface charge, size, lipid and protein composition as well as function. This physical and biochemical heterogeneity of HDLs reflects their perpetual interactions with a number of plasma factors which mediate changes in structure and cardioprotective proprieties of these lipoproteins. These events are collectively termed HDL remodeling.

The first classification of HDL was made according to their electrophoretic mobility: α, pre-α, pre-β and γ. β-HDLs have a discoidal shape and are referred to as “nascent”, while α-migration HDL are spherical particles and are commonly named “mature”. In turn, α-HDL can be subdivided into HDL2 (large HDL) and HDL3 (small and dense HDL). A further classification can be made by a more sophisticated and precise electrophoretic method: Lipoprint. This analytical method allows to separate lipoproteins on polyacrylamide gel and to identify 10 HDL subtypes. The 1–3 types are classified as large, 4–7 types as intermediate, and 8–10 types as small particles. The importance of this method relies on the fact that the antiatherogenic function of HDL appears to be inversely correlated with their size, with small-dense HDL subclasses being considered the most anti-atherogenic ones [26,27]. However, this remains controversial with large HDL particles being reported as beneficial for vascular health [28].

The heterogeneity in the HDL structure also affects their functionality, suggesting these two aspects of HDL biology being interrelated. In this regard, when referring to HDL functionality, the fact that the function of these lipoproteins span beyond the reverse transport of cholesterol in order to favor its biliary excretion must not be overlooked. Indeed, HDL is also able to induce nitric oxide synthesis, and to act as an anti-inflammatory, antioxidant and anti-apoptotic agent (Figure 1). Notably, some of the measures of these functional proprieties, in particular the ability of HDL to promote cholesterol efflux, are better predictors of CAD, but also dementia, independently of plasma levels of HDL-C [29,30,31].

As previously pointed out, the function of the HDL particle is driven by the composition in proteins and lipids, in particular phospholipids, which are the most abundant type in the lipoprotein. It was shown that the relative increase in phosphatidylcholine favors cholesterol efflux, while the same change in sphingomyelin decreases influx of cholesterol towards the liver [32]. Regarding the HDL proteome, it is mandatory to make a distinction between constitutive apolipoproteins and accessory enzymes. Apolipoprotein A1 (ApoA1) is the most abundant making up HDL lipoproteins (accounting for greater than 70% of the HDL-associated proteins) and functionally relevant apolipoprotein in HDL. This macromolecule is a key player in regulating cholesterol homeostasis. It activates lecithin cholesterol acyltransferase, and interacts with various other proteins involved in reverse cholesterol transport, such as ATP Binding Cassette AI and GI, and scavenger receptor BI [33]. It also serves as a structural scaffold that maintains lipoprotein stability and mediates the docking of a variety of accessory proteins, which remodel HDL throughout their lifespan. Apo AII is the second most abundant protein in HDL, and accounts for up to 20% of the protein in the HDL particle; it plays a pivotal role in HDL remodeling by increasing the stability and reducing the capacity of HDL to undergo conformational changes. Other relevant apolipoproteins are ApoE (present in various isoforms determined by correspondent genetic polymorphisms), ApoJ (also termed clusterin), ApoCI and ApoCIII. 

Lecithin cholesterol acyltransferase and cholesteryl ester transfer protein are well-known accessory proteins that enrich the complex mosaic of HDL. Besides these, there are other specialized enzymes that exert multiple functions and, thus, contribute to the well-recognized pleiotropic nature of these lipoproteins. The majority of them catalyze vasculo-protective activities, being implicated in systemic antioxidant and anti-inflammatory defensive mechanisms. The main member of this group are paraoxonase-1 (PON1), PON3, lipoprotein phospholipase A2, glutathione peroxidase-3. The loss of these proteins and/or enrichment of HDL particles with the pro-oxidant and pro-inflammatory myeloperoxidase and serum amyloid A contributes to the shift from functional to dysfunctional HDL.

### 4.2. Functional HDL

There is a wealth of evidence showing that in some diseases the atheroprotective function of HDL is lost and sometimes even inverted (Figure 1). It was shown that HDLs isolated from patients with CAD, diabetes and kidney disease have a decreased ability to stimulate nitric oxide production, promote cholesterol efflux from macrophages, restore endothelial function and protect lipids from oxidation. It is not completely understood whether this shift from functional to dysfunctional HDL is the cause or effect of the disease. The case of the HDL-associated PON1 is emblematic in this context. Reduction in PON1 activity and concentration has been shown in several diseases. PON1 mostly contribute to the capacity of HDL to protect LDL from oxidation occurring in the systemic circulation and into the sub-endothelial space. Thus, it is likely that the widely documented reduction in its activity reflects an increment in the pro-atherogenic oxidized LDL particles, thereby predisposing to CVD and CAD. However, in vitro experiments showed that PON1 is susceptible to oxidation (as well as glycation) which largely affects its catalytic efficiency [34]. Since systemic redox imbalance is a common pathophysiological feature of all the above mentioned disorders, it is difficult to discern if the fall in activity of PON1 is a downstream or upstream event in their pathogenesis.

To explain why very high levels of HDL-C were related to elevated risk of CAD in three large observational studies, authors hypothesized that a large fraction of these lipoproteins transitioned towards a pro-atherogenic phenotype [35,36,37]. This dramatic change in function may, in turn, be the consequence of a systemic state of chronic and acute inflammation and oxidative stress. It was demonstrated that serum amyloid A, produced in a large amount during acute-phase, interacts with HDL rendering these particles pro-inflammatory [38]. Similar results are yielded by myeloperoxidase. This heme-containing enzyme is released by monocytes, leukocytes and macrophages and is a well-documented biomarker of CAD. It is present in a relevant amount in the atherosclerotic plaques, where it catalyzes the oxidation of halides and pseudohalides in the presence of hydrogen peroxide (H_2_O_2_); the products of these reactions such as hypochlorous acid (HOCl), hypobromous acid, hypoiodous acid and hypothiocyanic acid are highly pro-oxidant molecules. The main targets of myeloperoxidase-generated reactive species are ApoA1 and PON1. This toxic activity seems to be facilitated by the fact that myeloperoxidase likely binds to HDL in close proximity to both proteins. The oxidation of HDL-embedded proteins has important repercussions on the overall functionality of these lipoproteins. In keeping with this, in vitro and in vivo studies showed that the oxidation of ApoA1 impairs ATP Binding Cassette AI-dependent cholesterol efflux from macrophages [39]. In particular, myeloperoxidase appears to mediate the oxidation of specific residues of ApoA1 which are implicated in triggering cholesterol efflux mechanisms [40].

Additionally, although the oxidation of ApoA1 increases the affinity for SR-B, it impairs the cholesterol efflux mediated by this receptor [39]. Moreover, oxidized apoA-I led to a nearly 90% reduction in lecithin cholesterol acyltransferase along with a decrease in the antioxidant capacity of HDL [41,42].

### 4.3. Evaluation of HDL Function

As already underlined, the assessment of HDL-C circulating levels does not necessarily reflect HDL functionality, which is the real causal factor of atherosclerosis. Thus, being able to objectively measure HDL function becomes a fundamental task in the clinical biochemistry field. Nevertheless, the complex pleiotropic effects of HDL make the development of a single measurement challenging [43]. In agreement with this technical challenge, at present, there is no “gold standard” technique for the measurement of HDL function. 

The currently available in vitro and ex vivo methods are multiple and range from the assessment of the functional capacity of HDL isolated from patients to the assessment of the activity of single lipoprotein-associated enzymes. From a functional prospective, the assessment of reverse cholesterol transport allows to evaluate both HDL–LCAT enzyme activity as well as the efflux of cholesterol from macrophages (radioactive or fluorescence assays). However, as already extensively discussed, the function of HDL is not limited to reverse cholesterol transport; therefore, the in vitro assessment of the antioxidant and anti-inflammatory capacity of isolated HDL provides further important insights into the functional status of the lipoprotein. 

Considering these proprieties being the result of the enzymatic actions of accessory proteins and their interaction with other components of HDL proteome and lipidome, it is also informative to measure the activity of PON1, glutathione peroxidase 3, myeloperoxidase etc., as well as the entire protein and lipid composition by well-validated mass spectrometry techniques combined with chromatographic techniques. Details on the assays for HDL function are out of the scope of this review, but have been exhaustively reviewed by Hafiane and Genest [43].

### 4.4. HDL-like Particles and Cerebrovascular Diseases

A wealth of epidemiological/clinical evidence suggest that low HDL-C level is associated with cognitive impairment due to increased carotid artery atherosclerosis (leading to cerebral hypoperfusion), damage of the BBB, microbleeds and small vessel disease [44]. However, as for CAD, there are also conflicting data casting some doubts about the causal link between HDL-C and CVDs [45]. In particular, some longitudinal studies have found an inverse relationship between HDL-C levels and stroke risk [46,47], while others have found no [48,49,50], or even positive association [51].

Once again, the possible explanation of these unexpected contradictory findings may be due to the fact that HDL-C level does not reflect the real protective proprieties of these lipoproteins. Despite this intriguing hypothesis, there are only a few studies addressing the impact of dysfunctional HDL on VaD. The available findings point to two possible mechanisms: (1) dysfunctional HDL increases the risk of atherosclerosis, which is the foremost risk factor for CVDs [52] and (2) dysfunctional discoidal HDL can cross the BBB, and thus may directly promote cerebral arteriopathy [53].

The only lipoproteins responsible for cholesterol (and phospholipid) transport in the brain are the so-called HDL-like particles (HDLlp). These still poorly characterized carriers are present in the cerebrospinal fluid (due to its accessibility, most investigations on the brain lipoproteins have been carried out on this fluid) at a level that is roughly 100 times lower compared to plasma HDL. Despite the evident gap in knowledge of brain HDL, it seems quite clear that its composition and structure are significantly different from that of the plasmatic counterpart. One of the most evident differences is regarding the proteome, with APOE being the major protein in HDLlp, while APOA1 is the most abundant in plasma HDL [54,55]. They also appear to differ for other apolipoproteins and accessory proteins as well as lipid composition, as extensively reviewed elsewhere [56].

HDLlp is also a heterogeneous family. The biogenesis and maturation of these particles are complex and still not completely understood. It has recently become apparent, however, that the metabolism of these particles involves pathways that are independent of the peripheral compartment. The same considerations can be applied for brain cholesterol, which is mostly synthesized de novo by glial cells and astrocytes. Indeed, the BBB represents an almost impermeable barrier for plasma lipoproteins. Nevertheless, HDL appear to be transported through the BBB via a saturable transport mechanism [54].

Nascent HDLlp have the same discoidal shape as their plasma counterpart, but while the latter is formed by apposition of free cholesterol and phospholipid to APOA1, the scaffold of the former is represented by APOE. This apolipoprotein is predominantly synthetized by astrocytes, and to a lesser extent by oligodendrocytes, microglia, ependymal layer cells and, under specific stress conditions, by neurons [55]. On the contrary, cerebrospinal fluid ApoA-I comes from the periphery and its uptake is mediated by SR-BI via the choroid plexus [57,58]. The mechanism underlying this transfer is still not completely understood, but mounting evidence suggests that it could, at least in part, be mediated by plasma discoidal HDL. Indeed, in vitro experiments demonstrated that this subclass of lipoproteins can cross the BBB, dragging to the brain its content, including APOA1 and accessory proteins such as PON1 [59]. Whether the other HDLlp apolipoproteins are generated in situ or come from the periphery is still uncertain. 

The large majority of HDLlp are spherical and contain also ApoJ, ApoA-II, ApoA-IV, ApoD and ApoH. This means that this lipoprotein is also subject to remodeling. The identification of key enzymes (e.g., lecithin cholesterol acyltransferase, cholesteryl ester transfer protein) and cholesterol/phospholipids transporters (scavenger receptor BI, LDL receptor, ATP Binding Cassette AI and GI) within the CNS supports the possibility that the process of HDLlp maturation takes place similarly to HDL in the periphery [58,59,60].

### 4.5. Dysfunctional HDLlp

There is a large and consistent body of evidence showing that excessive cholesterol accumulates in the brain leading to AD-associated pathological changes [61,62]. On the contrary, the implication of impaired brain cholesterol metabolism in VaD has been poorly investigated.

As occurs for HDL in the periphery, impairment in HDLlp function negatively affects cholesterol homeostasis in the brain. Since the cholesterol efflux capacity of these lipoproteins is positively correlated with ApoA-I, ApoE and ApoJ concentrations [63,64], structural and chemical modifications in these constituents may affect this fundamental function. The most solid proof of this hypothesis comes from the discovery of ApoE4 isoform as the strongest genetic risk factor for AD, but also other types of dementia including VaD [65]. Expression of this variant of ApoE is associated with an increase in LDL-C levels and increased risk of atherosclerosis [66]. Moreover, ApoE4 seems to lead to poor lipidation of HDLlp leading to lower efficiency in cholesterol efflux from astrocytes [58,67]. Further evidence uncovered that the accumulation of cholesterol in the brain promoted the formation of Aβ plaques and neurofibrillary tangles, the main neuropathological hallmarks of AD [61]. The genetic aspect is not the only determinant of APOE function. In line with this, ApoE is highly vulnerable both in the brain and the periphery to oxidative stress, which in concert with inflammation, represents a key feature of VaD. ROS-induced modifications of ApoE lead to structural changes and reduced lipid transport capacity [68]. This protein also acts as a sort of antioxidant scavenger. Indeed, it is able to bind 4-hydroxynonenal, the main secondary product of lipid peroxidation [69], thus preventing this reactive molecule from causing oxidative injury to neuronal proteins and possibly cell death. Interestingly, ApoE4 is less prone to form adducts than the other variants, and this could provide additional explanations accounting for its strong association with the risk of dementia [70].

As outlined above, the oxidation of APOA1 is one of the major causes of the shift from an athero-protective to an either ineffective or pro-atherogenic plasma HDL. Considering that apoA-I is the only known apolipoprotein of peripheral lipoprotein metabolism not expressed in the brain and that can cross the BBB, it is tempting to hypothesize that the functional change affecting this apolipoprotein could be reflected in HDLlp. The role of APOA1 in brain lipoproteins is still partly elusive. Indeed, it is still not clear whether and to which extent this protein participates, in combination with APOE, in HDLlp remodeling. However, plenty of studies showed that plasma APOA1-HDL correlate with cerebrospinal fluid APOA1 levels [71,72], and as a likely consequence of this relationship, a decrease in plasma and cerebrospinal fluid ApoA1 is associated with poorer cognitive performance and higher risk of having various forms of neurological diseases [62,73,74]. Importantly, the crossing of a modified ApoA1 through the BBB could explain why the capacity of promoting cholesterol efflux is impaired in both HDLlp and plasma HDL in AD patients [64].

HDL becomes dysfunctional also through the physical association with acute phase proteins, firstly serum amyloid A. Notably, although it is still not clear whether HDLlp also undergoes remodeling during acute or chronic inflammation, it has been demonstrated that serum amyloid A levels significantly increased in the cerebrospinal fluid of AD patients [75]. Moreover, in vitro studies showed that serum amyloid A can increase remodeling of cerebrospinal fluid HDL inducing apoE dissociation [76]. Whether this process leads to a reduction in HDLlp functionality remains to be determined.

## 5. Evidence from Observational Studies Linking HDL and HDLlp to VaD

A large body of evidence showed a close link between plasma dysfunctional HDL and the main risk factor of VaD, i.e., atherosclerosis (Figure 1). There are also direct clinical proofs indicating that a decrease in HDL functionality is associated with a higher risk of having CVD, in particular stroke [77]. However, the majority of the evidence supporting the role of HDL functionality in CVD have been generated by investigations on determinants of HDL antioxidant and anti-inflammatory activity, such as PON1, myeloperoxidase and Lp-PLa2 [72,78,79,80,81,82,83].

With regard to the anti-oxidant role of HLD, we and other researchers found that serum activity of PON1 is decreased in patients with VaD compared to cognitively healthy controls [72,78,79]. We have also found that lower PON1 activity in patients with mild cognitive impairment (prodromal stage of dementia) is associated with higher risk of developing VaD [84]. These findings corroborate those by Bednarz-Misa and coworkers who showed that a decrease in PON1 reflects a degree of brain ischemia [78]. Two recent studies suggest that PON1 is present in the brain and cerebrospinal fluid, pointing to a systemic origin of this protein [59,72]. Indeed, PON1 and PON3 protein expression was demonstrated in the brain despite no PON1 gene expression [59]. Moreover, the activities of serum and cerebrospinal fluid PON1 were strongly correlated [72]. In line with these findings, a metanalysis showed that the PON1 polymorphisms are associated with a small increase in the risk of ischemic stroke [85]. However, it cannot be ruled out that this alteration in PON1 levels is the result of a redox imbalance following stroke. Indeed, it is well-known that PON1 is highly vulnerable to oxidative modification. Thus, the pronounced oxidative damage found in HDL isolated from stroke patients could also contribute to the observed decrease in PON1 activity [86].

Relevant to this context, Ortiz-Munoz et al. found that HDL particles from patients affected by acute stroke are characterized by qualitative abnormalities, beyond the decrease in PON1 content [77]. Indeed, they present higher HDL content of myeloperoxidase and acute phase proteins. This abnormal proteome composition impaired the ability of HDL to inhibit TNF-induced expression of chemokines, cell adhesion molecules and matrix metallopeptidase 3 that compromise the function of brain microvascular endothelial cells, the major components of BBB [77]. These results were concordant with data from animal studies reporting that the intravenous injection of purified HDL in a rat model subjected to embolic occlusion exerted beneficial effects in terms of mortality, cerebral infarct volume, inflammatory response and BBB integrity [87].

Myeloperoxidase participate in the occurrence and development of stroke [82]. This enzyme is released in large amount by cells responsible for the innate immune response (in particular neutrophils) during inflammation. It has been suggested that myeloperoxidase is able to bind HDL, causing an impairment of PON1 and ApoA1 functionality. Myeloperoxidase can participate in the onset and exacerbation of stroke-induced damage. It damages the arterial and promote the formation of atherosclerotic plaques. The rupture of the BBB, occurring after stroke, makes the brain accessible to several immune cells, including neutrophils. The production of myeloperoxidase from these cells exacerbates the damage to the BBB and contributes to fuel neuroinflammation, even after stroke is resolved [88].

Lipoprotein phospholipase A2 is also bound to HDL (although the major carrier is LDL), and plays a role in atherosclerotic plaque inflammation and instability [57]. Expression of Lipoprotein phospholipase A2 is increased in carotid atherosclerotic plaques of patients with recent stroke or transient ischemic attack [89].

In spite of this large body of evidence, the direct link between dysfunctional HDL or HDLlp and VaD is still lacking, with no studies investigating the functional state of these lipoproteins in patients suffering from this form of dementia. 

### Mechanisms Linking Dysfunctional HDL and HDLlp and VaD

The large majority of studies exploring the potential role of HDLlp in dementia development are focused on AD. However, the findings of these investigations can be helpful in order to understand how these brain lipoproteins can be implicated in the onset and progression of VaD. Indeed, these two forms of dementia share several pathogenic and pathophysiological features. This overlap has been firstly suggested by epidemiological studies which showed that AD is also associated with vascular risk factors, such as hypertension, peripheral atherosclerosis and diabetes [90]. This body of evidence has subsequently found support from studies employing imaging techniques, showing that vascular abnormalities, such as large infarcts, lacunae and multiple microinfarcts, hemorrhage, atherosclerosis, and arteriolosclerosis are highly prevalent in AD [90,91,92]. Additional common neurovascular comorbidity observed in this form of dementia is cerebral amyloid angiopathy. This neurovascular abnormality is the result of the deposition of amyloid-β, i.e., the primary constituent of senile plaques in AD, in cerebral vessels [92]. It has been suggested that cerebral amyloid angiopathy might play an important pathogenic role in AD by affecting perivascular drainage, a major route of amyloid-β clearance from the brain, and thus favoring the accumulation of these aberrant peptides [93]. Intriguingly, an increase in brain cholesterol appears to contribute to the impairment of Aβ clearance and deposition of Aβ in the vascular wall [94]. Notably, a recent study revealed that AD patients with cerebral amyloid angiopathy have plasma HDL which differ in protein and lipid composition from those of AD without the angiopathy [95].

A further common feature of AD and VaD is the prominent role of oxidative stress as a pathogenic mechanism. Accordingly, increased levels of biomarkers of oxidative damage to lipids (especially isoprostanes), proteins (nitrotyrosine and carbonlyles) and nucleic acids (8-deoxyguanosine) have been detected in the brain of patients affected by either of the diseases [96,97,98]. In AD, an excessive increase in reactive species has been widely referred to as an early pathogenic event [96]. ROS are generated in great amounts by microglia, astrocytes and endothelial cells by NADPH oxidase, mitochondria and xanthine oxidase in response to cellular insults, including ischemic and hemorrhagic brain injury following a cerebrovascular event [99]. An oxidative environment has many detrimental effects on cerebral vessels, mostly due to the impairment of endothelium-dependent nitric oxide signaling and the decrease in this potent vasodilator [100]. Owing to these considerations, the oxidative modification of HDLlp and relative consequence on biological properties of these lipoproteins is a likely scenario in both diseases.

Owing to the, although still incomplete, current state of knowledge on the structure, composition and function of HDLlp and similarities with HDL, it can be hypothesized that similar perturbations also tackle these CNS lipid carriers. In turn, these changes may affect their capacity to mediate cholesterol homeostasis, and in general their putative pleiotropic nature. Indeed, although it is still unknown if HDLlp maintains the multiple athero-protective roles of plasma HDL, it is well-established that many of the functional determinants (e.g., ApoA1, ApoE, PON1, PON3 etc.) of the latter are conserved in the former. Moreover, in vivo studies demonstrated that modulation of some of these factors has direct consequences in the brain [101]. In particular, it was demonstrated that overexpression of APOA1 attenuates whereas its deficiency exacerbates cerebral amyloid angiopathy [101]. 

Regardless of the mentioned gap in knowledge, HDLlp certainly has a key role in preventing cerebral atherosclerosis, one of the main etiological factors of cerebrovascular disease and related dementia [52]. This condition is characterized by cerebrovascular lipid deposition and the disease of large, medium, and small vessels, including arteriosclerosis [52], which is a direct consequence of impaired cholesterol efflux and influx by HDLlp. In keeping with this, as indirectly demonstrated by several epidemiological/clinical studies linking the APOe4 variant with cerebral atherosclerosis, a dysfunctional APOe can compromise this function also in the CNS [52]. 

## 6. Ceramides as a Novel Potential Driver of Cerebrovascular Disease

**Figure 2:**
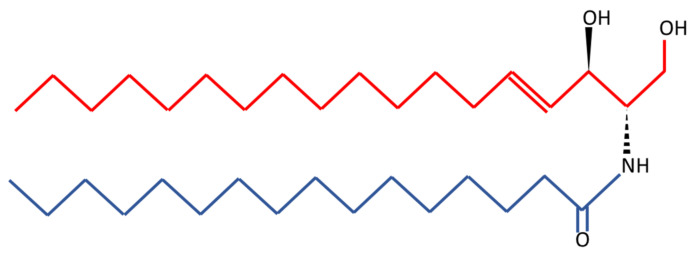
C16 ceramide (d18:1/16:0) structure. Ceramides are composed by a variable-length fatty acid chain (blue) and a sphingosine base (red). In the case of a C16 ceramide, the fatty acid chain is characterized by 16 carbons.

As already mentioned, another potential explanation of the residual risk, when the circulating lipid profile is in appearance healthy, is represented by the quality of the lipids contained within lipoproteins. A key example in this context is represented by ceramides, which in the circulation travel, at least in part, packed in lipoproteins, including HDL [102]. Ceramides are the precursors of complex sphingolipids including sphingomyelins, glucosylceramides and sphingosine, and are virtually produced in every cells via three different pathways: de novo synthesis, the sphingomyelinase pathway and the salvage pathway as extensively reviewed elsewhere [103]. Structurally, ceramides are highly hydrophobic molecules encompassing a fatty acid chain of variable length and a sphingosine base [9] (Figure 2). At the cellular level, ceramides are important components of the plasma membrane of which they are integral components as part of lipid rafts that play a key role in stabilizing the structure of cell membranes and the distribution of surface receptors [104]. Beside their structural role, ceramides are bioactive lipids able to modulate intracellular signaling pathways related to cell growth, apoptosis, mobility, differentiation and senescence [105,106,107]. The ability of ceramides to modulate such a wide array of cellular processes is a direct consequence of their ability to function as second messengers and target kinases such as jun kinases, kinase suppressor of Ras and the atypical protein kinase C zeta, as well as phosphatases such as protein phosphatase 1 and protein phosphatase 2A [107]. The modulation of these kinases and phosphatases culminates in the inhibition of pro-growth while augmenting pro-apoptotic signaling [107,108]. In light of their role as bioactive lipids, it follows that dysregulation of their metabolism may negatively impact upon human health. Indeed, the accumulation in the circulation of certain ceramide species, such as C16.0, C18.0 and C24.1, has been shown to promote CAD as well as insulin resistance [7,102,105,109,110,111,112]. In particular, ceramides have been linked with atherosclerosis progression and pathology [9,10] supporting a putative role of ceramides also in CVDs (Figure 1). Not surprisingly, ceramide levels have been reported to be increased in the brain of patients suffering from neurodegenerative diseases [113]. Additionally, the dysregulation of ceramide metabolism has been increasingly associated with neurodegenerative diseases and proposed as an upstream event in disease development [108]. However, although this association is plausible, it remains to elucidate whether the effects of ceramides on neurodegenerative diseases depends upon CVDs. Nevertheless, ceramides remain a promising class of lipids in promoting CVDs, also in light of their ability to trigger inflammatory responses [114], which in turn has been identified as a pathogenic factor in CVDs. Finally, independently of whether the ability of ceramides to promote CVDs is related to their involvement in the pathogenesis of atherosclerosis, or their ability to trigger inflammation, this class of lipids remains associated with CVDs. In keeping with this, higher levels of ceramide C16:0 and the ratios between ceramides C16:0/C24:0 as well as C24:1/C24:0 are associated with higher white matter hyperintensity volume which, in turn, is a marker of CVDs [115]. 

## 7. Dietary Lipids in the Pathogenesis of Cerebrovascular Disease

### 7.1. Omega-3 Fatty Acids

Dietary lipids have been shown to affect cognitive function and the risk of developing dementia [116,117]. In this regard, unsaturated fatty acids, and particularly the omega-3 fatty acids eicosapentaenoic acid (EPA) and docosahexaenoic acid (DHA), have been associated with a lower risk of all cause-dementia, including VaD [118,119,120]. In terms of the effects of omega-3 on VaD, these may depend upon the ability of these polyunsaturated fatty acids to preserve brain vasculature and improve cardio-metabolic health leading to a decrease in blood pressure, plasma triglycerides and an improvement in endothelial function [121,122,123]. Furthermore, omega-3 polyunsaturated fatty acids have been shown to reduce microinfarct burdens in rodents, further supporting the putative role of these dietary lipids in the prophylaxis and treatment of VaD [124]. In further support of this, an inverse relationship between CVDs and omega-3 fatty acid intake has been reported [125], which is in line with the notion that the beneficial effect of omega-3s on cognitive function appears to be limited to VaD and not AD [120]. Indeed, despite AD and VaD sharing similar pathogenetic mechanisms, VaD is predominantly triggered by cardiovascular dysfunction [126]; whereas, there is only a weak association between cardiovascular risk and AD [127]. Thus, considering the impact of omega-3 polyunsaturated fatty acids on cardiovascular health [128], it is not surprising that these dietary lipids appear to exert a protective effect on VaD and not on AD. 

**Figure 3:**
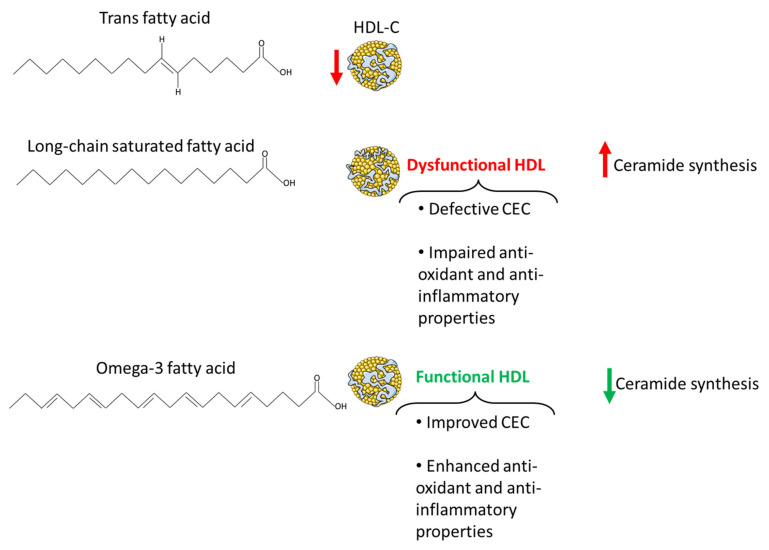
The effect of different dietary lipids on ceramide synthesis and HDL lipoproteins. Trans saturated fatty acids promote a decrease in circulating HDL-cholesterol. Long-chain saturated fatty acids, such as palmitic acid, impair HDL lipoprotein cholesterol efflux capacity, anti-oxidant and anti-inflammatory capacity, whereas the functionality of these lipoproteins is enhanced by omega-3 fatty acids. Finally, while palmitic acid fosters ceramide synthesis, this effect is countered by omega-3 fatty acids such as EPA. This figure was created using smart.servier.com.

In this regard, the beneficial effects of EPA and DHA on cardiovascular health also rely on the impact of these fatty acids on HDL lipoproteins, both in terms of their circulating levels as well as their quality and function. Increasing the intake of omega-3s has been shown to increase HDL2 [129,130], with these effects appearing to be particularly driven by DHA [13,131]. In terms of HDL quality and functionality, omega-3 fatty acids increased large while lowering small HDL and the amount of non-esterified fatty acids within these lipoproteins. Additionally, supplementation of fish oils, a source of omega-3s, may increase the anti-oxidant properties of HDL, but this remains controversial with some studies reporting an increase in PON1 content and HDL antioxidant function [132,133,134], while others not reporting an increase in HDL antioxidant potential [14]. Finally, HDL derived from patients supplemented with EPA were characterized by an improvement in their anti-inflammatory effects along with an enhancement of their capacity to mediate cholesterol efflux [134] (Figure 3). 

Additionally, the beneficial effects of omega-3 fatty acids on CVDs may also rely upon their ability to modulate lipid metabolism, thereby lowering ceramide synthesis. Despite this, evidence in support of this notion in humans is limited and to some extent controversial. Indeed, while some reports provided evidence on the ability of these polyunsaturated fatty acids to lower plasma ceramides [135], others failed to report the same effect upon omega-3 fatty acid supplementation [136]. More consistent are the data arising from animal and cell models. In keeping with this, DHA and EPA have been reported to inhibit palmitic acid-induced ceramide synthesis in a variety of cell models, including myotubes [137], hypothalamic neurons [16] and macrophages [138] (Figure 3). To the same extent, in animal models, EPA and DHA supplementation led to a decrease in ceramide levels in skeletal muscle [139], liver [140] and adipose tissue [141].

Thus, taken together, these findings suggest that the beneficial effects of omega-3 fatty acid supplementation upon CVDs may rely upon the modulation of HDL quality and functionality as well as ceramide metabolism.

### 7.2. Long-Chain Saturated Fatty Acids

Long-chain saturated fatty acids, such as palmitic acid, as opposed to omega-3 fatty acids were positively associated with VaD along with other types of dementia [142]. Reduced brain perfusion, oxidative stress, neuroinflammation, disruption of the cerebrovascular epithelial cells as well as insulin resistance are all key mechanisms underpinning the effect of saturated fatty acids overconsumption on cognitive function [143,144]. However, atherosclerosis potentially represents the primary link between saturated fatty acids and VaD [145]. The effects of diets rich in saturated fatty acids on CVDs are strictly related to the cardio-metabolically deleterious effects of these dietary lipids, as extensively reviewed elsewhere [143]. Nevertheless, controversies still exist with regard to the role of saturated fatty acids on cardiovascular health. In this regard, recent evidence suggests that the detrimental effects of saturated fatty acids are dictated by the food matrix they are embedded in. Indeed, when saturated fatty acids are consumed as part of unprocessed as opposed to processed foods, they are not associated with an increased risk of developing CAD or diabetes [146]. This paradigm may also apply to HDL circulating levels and functionality, pointing towards HDL quantity and quality as further discriminants linking saturated fatty acids overconsumption with CVDs and VaD. In line with this, saturated fatty acids themselves do not appear to negatively impact upon HDL circulating levels, instead they appear to increase the plasma levels of these lipoproteins [13]. This effect appears to be fatty acid-dependent, as it is particularly evident for lauric acid which was shown to induce a decrease in the ratio of total to HDL cholesterol [147]. However, there is also evidence that the long-chain saturated fatty acid stearic acid lowers HDL-cholesterol relative to linoleic acid [148]. Additionally, palmitic acid was reported to increase LDL-cholesterol [149] with little or no effect on the total to HDL cholesterol ratio [147], suggesting that not all saturated fatty acids have the same impact on the development of CAD and consequently on CVDs and VaD. Nonetheless, the fact that saturated fatty acids are able to raise HDL-cholesterol is in disagreement with their impact upon HDL functionality. In support of this, a high-fat meal rich in saturated fatty acids impaired the anti-inflammatory properties of HDL lipoproteins, which were less effective in downregulating both intercellular adhesion molecule-1 and vascular cell adhesion molecule-1 in human umbilical vein endothelial cells [150] (Figure 3). Always in support of the different effect elicited by different saturated fatty acids, their quality also affects HDL-mediated cholesterol efflux as indicated by the ability of saturated fatty acids from butter, but not from cheese, to increase this HDL functional aspect [151]. Additionally, low-grade chronic inflammation, as already discussed, is another key player in shaping HDL cholesterol efflux capacity. Inflammation induces changes in HDL composition, marked by an increase in free cholesterol and triglycerides and a drop in phospholipids [152], and impairs HDL cholesterol efflux capacity [153]. Remarkably, saturated fatty acids and particularly long-chain saturated fatty acids have been widely demonstrated to trigger inflammatory responses in a variety of tissues [154,155,156,157,158], suggesting that their negative affect on HDL functionality may also be related to their ability to induce sterile inflammation. 

Ceramides also represent a potential mediator of the impact of saturated fatty acids on CVDs and VaD. Not surprisingly, ceramide synthesis is fostered by saturated fatty acids, and particularly by the long-chain saturated fatty acid palmitic acid [16,159] (Figure 3). Indeed, the latter represents a key building block for the synthesis of the ceramides via the de novo synthesis pathway [160]. Thus, considering the role of ceramides in the pathogenesis of atherosclerosis and CVDs, these sphingolipids represent an additional player underpinning the beneficial effects of lowering long-chain saturated fatty acid intake on cerebrovascular and cognitive health.

### 7.3. Trans Fatty Acids

Despite not all saturated fatty acids being shown to modulate HDL lipoprotein function and the ratio of total to HDL cholesterol, there are other dietary lipids, also abundant in highly processed foods, which have been consistently associated with a decrease in HDL cholesterol and an increase in cardiovascular risk: trans fatty acids. These fatty acids are well known for their pro-atherogenic properties. Indeed, beside their ability to increase small and dense LDL-C, they are also able to lower HDL-cholesterol [148,161], also relative to saturated fatty acids [162]. In terms of CVDs, individuals who restricted the intake of trans fatty acids experienced a tendency towards a decline in stroke [163], suggesting a role of these fatty acids in cerebrovascular health and possibly VaD. However, despite their involvement in the pathogenesis of CAD, and their deleterious effects on cognitive function as well as association with AD risk [164], there is limited evidence with regard to the direct impact of trans fatty acids on VaD [142].

## 8. Conclusions

HDL lipoproteins and particularly their functionality as well as circulating ceramides travelling in the bloodstream as part of lipoproteins may represent promising targets to tackle CVDs with consequent beneficial repercussions on VaD. Additionally, both HDL functionality and ceramide metabolism appear to be nutritionally modulated, with omega-3 saturated fatty acids improving HDL functionality and lowering ceramide synthesis as opposed to long-chain saturated and trans fatty acids. Nevertheless, despite HDL functionality and ceramides representing promising pharmaceutical and nutritional targets to improve CVDs, further studies are warranted in order to confirm the therapeutical importance of HDL lipoproteins and ceramides in CVDs and consequently VaD. 

## Data Availability

Not applicable.
